# Evaluation of Transfer Learning Efficacy for Surgical Suture Quality Classification on Limited Datasets

**DOI:** 10.3390/jimaging11080266

**Published:** 2025-08-08

**Authors:** Roman Ishchenko, Maksim Solopov, Andrey Popandopulo, Elizaveta Chechekhina, Viktor Turchin, Fedor Popivnenko, Aleksandr Ermak, Konstantyn Ladyk, Anton Konyashin, Kirill Golubitskiy, Aleksei Burtsev, Dmitry Filimonov

**Affiliations:** 1 V.K. Gusak Institute of Emergency and Reconstructive Surgery, 283045 Donetsk, Russia; mxsolopov@gmail.com (M.S.); turchin.dn@mail.ru (V.T.); popfedor@list.ru (F.P.); doctor.ermak@yandex.ru (A.E.); dfh346@rambler.ru (K.L.); ant0n.konyashin@yandex.ru (A.K.); kirill.golubitskiy@mail.ru (K.G.); burtsevoleksiy@mail.ru (A.B.); 2 Medical Research and Educational Institute, Lomonosov Moscow State University, 119234 Moscow, Russia; voynovaes.pharm@gmail.com

**Keywords:** transfer learning, convolutional neural networks, image classification, surgical sutures, surgical skill assessment, machine learning, medical imaging

## Abstract

This study evaluates the effectiveness of transfer learning with pre-trained convolutional neural networks (CNNs) for the automated binary classification of surgical suture quality (high-quality/low-quality) using photographs of three suture types: interrupted open vascular sutures (IOVS), continuous over-and-over open sutures (COOS), and interrupted laparoscopic sutures (ILS). To address the challenge of limited medical data, eight state-of-the-art CNN architectures—EfficientNetB0, ResNet50V2, MobileNetV3Large, VGG16, VGG19, InceptionV3, Xception, and DenseNet121—were trained and validated on small datasets (100–190 images per type) using 5-fold cross-validation. Performance was assessed using the F1-score, AUC-ROC, and a custom weighted stability-aware score (Score_adj_). The results demonstrate that transfer learning achieves robust classification (F1 > 0.90 for IOVS/ILS, 0.79 for COOS) despite data scarcity. ResNet50V2, DenseNet121, and Xception were more stable by Score_adj_, with ResNet50V2 achieving the highest AUC-ROC (0.959 ± 0.008) for IOVS internal view classification. GradCAM visualizations confirmed model focus on clinically relevant features (e.g., stitch uniformity, tissue apposition). These findings validate transfer learning as a powerful approach for developing objective, automated surgical skill assessment tools, reducing reliance on subjective expert evaluations while maintaining accuracy in resource-constrained settings.

## 1. Introduction

The quality of surgical suture placement is one of the most critical factors determining successful surgical outcomes and patient safety. Errors in suturing or anastomosis formation can lead to serious complications, such as dehiscence, infection, hemorrhage, and stenosis. Studies indicate a high incidence of complications related to suturing technique and material choice. For example, in penetrating keratoplasty, suture-related complications such as erosions, infiltrates, and suture loosening occur in 2% to 11% of cases [1,2,3]. The influence of suture material choice on complication rates has also been demonstrated in studies on laparotomy wound closure [4,5]. Overall, surgical complications remain at a high level (over 11%), underscoring the need for continuous improvement in surgical techniques and their assessment methods [6].

Crucially, the precision of surgical anastomosis formation is paramount for patient safety and recovery, extending far beyond technical execution to directly impact morbidity and mortality. Errors in anastomotic technique can precipitate catastrophic complications, including anastomotic leakage [7], hemorrhage [8,9], strictures [10,11], abscess formation, and ileus [12,13]. These complications significantly impede recovery, prolong hospitalization, necessitate re-interventions, and substantially increase healthcare costs. For instance, anastomotic leakage alone is associated with high mortality rates, sepsis, and delayed return to function [7,14]. Optimal anastomotic technique—tailored to anatomy, tissue type, and tension—directly mitigates these risks, fostering secure tissue healing, reducing infection rates, and enabling faster functional recovery [12,15]. Consequently, objective assessment of anastomotic quality is not merely a procedural concern but a fundamental determinant of early patient recovery and long-term surgical success.

Traditional suture assessment relies on subjective expert evaluation [16,17], which lacks standardization and scalability. Recent advances in machine learning offer objective, automated alternatives. Various approaches utilizing the analysis of images, video recordings, kinematic data, and force parameters show promise for the quantitative assessment of technical aspects of surgical procedures [17,18,19,20,21,22,23,24,25]. Particular attention has been given to methods for assessing suture quality based on the analysis of final result images. The application of convolutional neural networks (CNNs) and other deep learning methods has led to successful automatic classification of suture quality from photographs. For instance, using the Xception architecture, 95% accuracy was achieved in distinguishing between successful and unsuccessful sutures [26]. Systems have been developed for the automatic extraction of geometric suture characteristics from images [18] and for assessing the effectiveness of microsuture placement [27], which demonstrated good correlation with expert assessments and physical measurements.

However, a major challenge in implementing machine learning-based methods in medicine is the scarcity of annotated data. The collection and annotation of large datasets of surgical suture images require considerable resources and expert time. Transfer learning addresses this challenge by leveraging pre-trained models on large datasets (e.g., ImageNet) and adapting them to small-scale medical tasks. This approach reduces the need for extensive annotated data while maintaining accuracy. Transfer learning with pre-trained models reduces data requirements and accelerates the training process while maintaining high classification accuracy [28,29,30,31].

This study addresses several key limitations previously identified in the current literature. First, we present a novel systematic comparative analysis of eight state-of-the-art CNN architectures on real ex vivo datasets representing three anatomically and technically distinct suture types. This provides insights into model performance in diverse surgical contexts. Second, we propose a composite performance metric that accounts not only for classification quality but also for inter-fold variability, thereby enabling more robust model selection in low-data conditions—a significant advancement for practical applications with limited annotations. Third, we demonstrate the feasibility and strong effectiveness of transfer learning for surgical image classification under realistic data constraints, showing that high performance can be achieved without large-scale annotation efforts. Finally, we apply GradCAM-based interpretability analysis to verify that the models focused on clinically meaningful visual features, supporting their potential integration into objective, explainable skill assessment tools.

## 2. Materials and Methods

### 2.1. Dataset Formation and Annotation

Datasets of three types of surgical sutures were created for the study: 105 images of continuous over-and-over open sutures (COOS), 100 images of interrupted laparoscopic sutures (ILS), and 380 images of interrupted open vascular sutures (IOVS) (190 external and 190 internal views). The distribution of high-quality (class 1) and low-quality (class 0) surgical suture images was as follows: external IOVS—115 high-quality, 75 low-quality; internal IOVS—100 high-quality, 90 low-quality; COOS—31 high-quality, 74 low-quality; and ILS—43 high-quality, 57 low-quality. This indicates a moderate class imbalance, particularly in the COOS dataset, which was addressed during training using class-weighted loss.

Suturing and anastomosis formation were performed by surgeons of varying qualifications, including residents and medical students. The procedures were conducted on ex vivo porcine vascular tissues and intestinal segments obtained from a licensed abattoir. These tissues were selected for their biomechanical similarity to human anatomical structures.

Two abdominal and two vascular surgeons, each with at least 10 years of clinical experience, independently annotated the images. Each image was classified as “high-quality suture” or “low-quality suture”. Criteria for a high-quality suture included stitch uniformity, absence of visible tissue deformation, and tight apposition of tissues. Low-quality sutures included those with signs of wound edge separation, uneven thread tension, or tissue ischemia. In cases of disagreement in assessment, a third expert surgeon was involved to make the final decision. To quantify inter-rater reliability, Cohen’s kappa coefficient (κ) was calculated for each suture type dataset using the initial independent annotations from the two primary surgeons, prior to third expert adjudication.

### 2.2. Model Training Methodology

The following modern CNNs architectures were used for binary classification of surgical suture quality: EfficientNetB0, ResNet50V2, MobileNetV3Large, VGG16, VGG19, InceptionV3, Xception, and DenseNet121. Transfer learning was implemented by initializing the models with weights pre-trained on the ImageNet-1K dataset [32]. This allowed features learned from recognizing thousands of object types to be used as a basis for analyzing surgical sutures. To preserve these pre-learned patterns and prevent their overwriting during the initial stages, the initial layers of the neural networks were frozen. Before being input to the base model, images underwent architecture-specific preprocessing: resizing to 224 × 224 pixels for EfficientNetB0, ResNet50V2, MobileNetV3Large, VGG16, VGG19, DenseNet121, and 299 × 299 pixels for InceptionV3 and Xception, followed by conversion into numerical arrays using NumPy v2.2.4. Real-time data augmentation techniques were applied to increase the volume and diversity of the training set, including random horizontal flips, rotations, scaling, and contrast adjustments.

We trained models in two stages. In the first stage, only the added classification layers (a fully connected layer with ReLU activation and L2 regularization, dropout layers, and an output layer with a sigmoid activation function) were trained, with the base model weights frozen. In the second stage, fine-tuning was performed, which involved unfreezing some layers of the base model (the number of layers depended on the model architecture) and continuing training with a lower learning rate. The Adam optimization algorithm was used. The loss function was binary cross-entropy. To address class imbalance, a weighted loss function was applied, assigning a higher weight to errors on the minority class in inverse proportion to its frequency.

### 2.3. Model Quality Assessment

To reliably estimate model generalizability, particularly given the limited dataset size, stratified 5-fold cross-validation was employed. This standard approach provides an effective bias–variance trade-off for performance estimation. Using five folds ensures that the validation set (20% of data) is sufficiently large for stable evaluation, while the training set (80%) remains substantial for effective model learning. Data were randomly partitioned into five folds, preserving the original class distribution through stratification. Each model was trained five times, each time using four folds for training and one for validation.

The training process was monitored with the ModelCheckpoint callback, which saved model weights with the highest validation accuracy, and EarlyStopping, which halted training if validation errors did not improve for five epochs to prevent overfitting. Classification performance on each validation fold was assessed using standard metrics (Table 1): precision, recall, and F1-score [33]. Additionally, the ROC curve was analyzed with calculation of the area under it (AUC-ROC), and the optimal classification threshold was determined through an iterative search maximizing the F1-score on the validation set. Final results for each architecture were averaged over the five cross-validation folds, and the mean values and standard deviations of the metrics were calculated. For model interpretation, GradCAM heatmaps were generated and visualized to highlight the image regions most important for the model’s decision-making.

**Figure 1:**
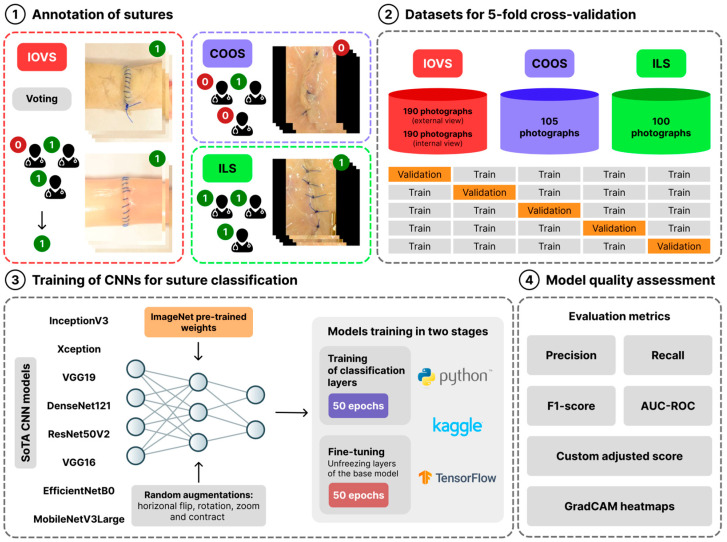
General study design. The process begins with annotating three types of sutures (IOVS—interrupted open vascular suture, COOS—continuous over-and-over open suture, ILS—interrupted laparoscopic suture), which are then used to train eight different state-of-the-art (SoTA) CNN models using a 5-fold cross-validation approach. The models are trained in two stages using transfer learning and data augmentation, and their performance is rigorously evaluated with standard metrics, a custom score, and GradCAM heatmaps.

To formally assess performance differences between the models, we employed the non-parametric Kruskal–Wallis H-test on the F1-scores obtained from the 5-fold cross-validation for each suture type. This test was chosen due to the small sample size per group (*n* = 5). A *p*-value of less than 0.05 was considered statistically significant. Additionally, the optimal neural network architecture for classifying a specific suture type was determined through 5-fold cross-validation, considering the average performance and model stability. For each architecture, a weighted performance score (*Score_fold_*) was calculated, aggregating key classification metrics using the formula:

(1) $${Score}_{fold}=\sum_{i=1}^{5}W_{i}\cdot {Metric}_{i,\;fold},$$

where *Metric_i_*_,*fold*_ is the value of the *i*-th metric on a given fold; *W_i_* is the weight coefficient of the metric (W_Accuracy_ = 0.10, W_Precision_ = 0.20, W_Recall_ = 0.20, W_F1−score_ = 0.35, W_AUC−ROC_ = 0.15). To account for the stability of the results, an adjusted score (*Score_adj_*) was used:

(2) $${Score}_{adj}={Score}_{mean}-\mu \cdot \;\sigma_{Score},$$

where *Score_mean_* is the mean value of the weighted score across folds; *σ_Score_* is the standard deviation of the weighted score; and *μ* is a coefficient to adjust the degree of influence of variability on the final assessment, with a value of 0.5 used. All computations and visualizations were performed using the Python v3.11 programming language with the following libraries: TensorFlow v2.18.0, scikit-learn v1.2.2, pandas v2.2.3, NumPy v2.2.4, Seaborn v0.13.2, SciPy v1.15.2, and Matplotlib v3.10.1. Model training and validation were conducted on the Kaggle web platform, utilizing an NVIDIA T4 GPU to accelerate the computational process. The overall study design is illustrated in Figure 1.

## 3. Results

### 3.1. IOVS Classification

**Figure 2:**
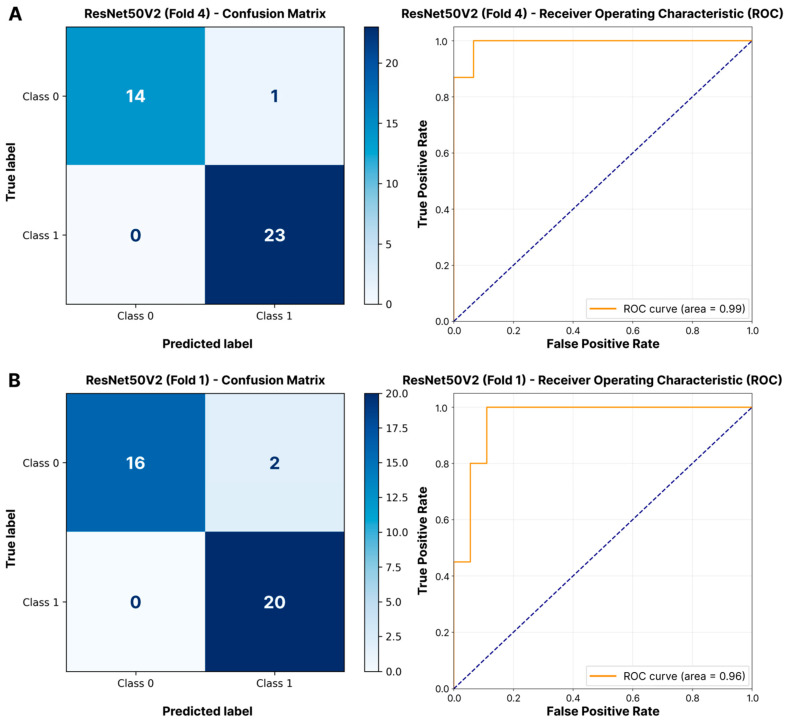
Classification diagnostics for IOVS. Confusion matrix and ROC curve for the optimal model (ResNet50V2) on the highest-scoring validation fold of (**A**) external and (**B**) internal views. Confusion matrices quantify errors between low-quality (class 0) and high-quality (class 1) suture categories, while ROC curves demonstrate threshold-dependent discriminative performance.

Statistical analysis revealed no significant difference in F1-scores among the evaluated models for either external (Kruskal–Wallis test, *p* = 0.60) or internal views (*p* = 0.23). As shown in Table 2, Xception achieved the highest F1-score (0.939 ± 0.033) for external IOVS classification, closely followed by ResNet50V2 (0.932 ± 0.031). MobileNetV3Large also showed a high F1-score (0.932 ± 0.033). The high AUC-ROC values (>0.90) indicate strong discriminative ability across suture types, with ResNet50V2 achieving the highest AUC-ROC (0.946 ± 0.025) for external IOVS classification. Based on the adjusted weighted performance score (Score_adj_), ResNet50V2 ranked first (0.915), closely followed by Xception (0.913) and MobileNetV3Large (0.911). For the classification of the internal view of IOVS, ResNet50V2 showed the best F1-score (0.931 ± 0.011) and also led in AUC-ROC (0.959 ± 0.008). The VGG16 model ranked second by F1-score (0.927 ± 0.022), and Xception ranked third (0.922 ± 0.033). By the Score_adj_ value, ResNet50V2 took first place (0.930), followed by VGG16 (0.918) and Xception (0.904). Visual confirmation (confusion matrices/ROC curves) of the high performance of the ResNet50V2 model for both IOVS views is presented in Figure 2. The confusion matrices and ROC curves for the best-performing validation fold demonstrate its strong discriminative ability.

### 3.2. COOS Classification

**Figure 3:**
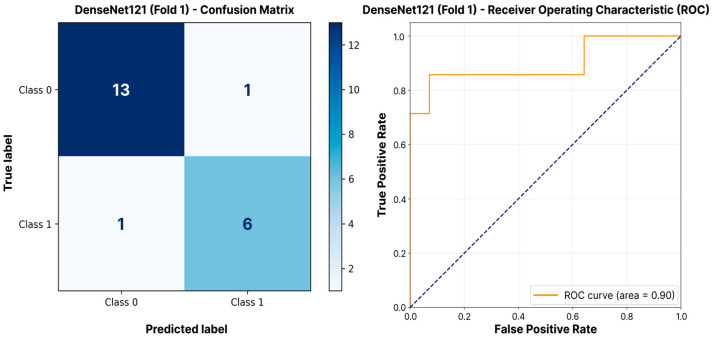
COOS classification performance. Confusion matrix and ROC curve for DenseNet121 (optimal model per Table 3) on highest-scoring fold.

The model performance evaluation results for COOS classification are presented in Table 3. The Kruskal–Wallis test showed no statistically significant difference in F1-scores among the architectures (*p* = 0.67). The DenseNet121 architecture demonstrated the best F1-score (0.788 ± 0.071) and AUC-ROC (0.853 ± 0.080). Xception and EfficientNetB0 models showed comparable but slightly lower F1-scores (0.740 ± 0.153 and 0.734 ± 0.055, respectively). Although the VGG16 model achieved high accuracy (0.925 ± 0.150), its recall was significantly lower (0.619 ± 0.142), resulting in a reduced F1-score (0.716 ± 0.078). Ranking of models based on the adjusted weighted score (Score_adj_), which considers both average performance and stability across folds, showed that DenseNet121 maintained first place (0.787), followed by EfficientNetB0 (0.746) and MobileNetV3Large (0.745). Figure 3 illustrates the performance of the leading DenseNet121 architecture on its best-performing validation fold, demonstrating that the model can achieve a high quality of classification (AUC-ROC = 0.90) even for this challenging suture type.

### 3.3. ILS Classification

**Figure 4:**
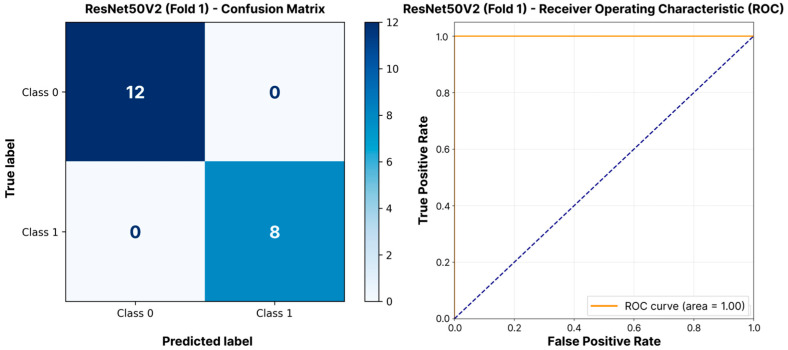
Confusion matrix and ROC curve for ILS classification using ResNet50V2 (best model per Table 4) on the optimal validation fold.

For ILS classification, all models showed significantly higher results (Table 4). We found no statistically significant difference in F1-scores across the models (Kruskal–Wallis test, *p* = 0.69). ResNet50V2 achieved the highest F1-score (0.950 ± 0.047), demonstrating perfect precision (1.000 ± 0.000) and high recall (0.908 ± 0.084). The VGG16, DenseNet121, EfficientNetB0, and MobileNetV3Large architectures showed very similar F1-scores (>0.939). EfficientNetB0 had the highest AUC-ROC (0.971 ± 0.024), indicating excellent discriminatory power. In the final ranking, ResNet50V2 also took first place by Score_adj_ (0.931), slightly ahead of EfficientNetB0 (0.920) and MobileNetV3Large (0.919). The exceptional performance of the ResNet50V2 model is clearly demonstrated in Figure 4. The confusion matrix for the optimal fold shows flawless classification, while the ROC curve, with an area of 1.00, confirms the model’s perfect discriminative power.

### 3.4. Model Interpretation

**Figure 5:**
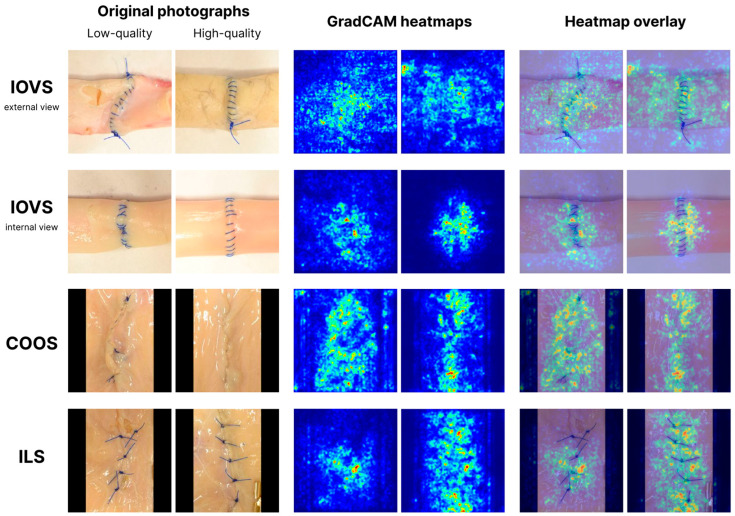
GradCAM heatmap examples demonstrating the image regions most attended by CNN models during surgical suture quality classification.

To analyze the image regions that the models focused on during classification, GradCAM heatmaps were generated. Examples of heatmaps for the identified best models for classifying each suture type are presented in Figure 5. GradCAM visualizations reveal model focus areas: stitches, knots, and tissue edges. These regions align with surgical quality assessment criteria.

## 4. Discussion

This study assessed the efficacy of transfer learning for classifying surgical suture quality from images. The results demonstrate the high potential of this approach, achieving robust classification performance on limited annotated datasets (from 100 to 190 images per class). Across all four classification tasks, ImageNet-pretrained models fine-tuned on the target data achieved high F1-scores, often exceeding 0.90 for IOVS and ILS (Table 2 and Table 4). This supports the hypothesis that features extracted by CNNs from general images can be effectively adapted for the analysis of specific medical images, such as surgical sutures, which is consistent with previous work in this field [26,34].

Notably, our statistical analysis did not reveal significant differences in F1-scores among the top-performing models for any of the suture types. This highlights a common scenario in which multiple advanced architectures achieve a similarly high performance, making selection based on a single metric challenging. Consequently, the Score_adj_ metric became critical by providing a more comprehensive evaluation. It rewards both model stability and broader discriminative power (AUC-ROC), which are paramount for reliable model selection in data-constrained medical imaging tasks. The use of the Score_adj_ metric, which penalizes models for high variability in results across cross-validation folds, enabled a more reliable ranking of model performance. This approach favored models that demonstrated not only high average metrics but also stable performance. For example, although Xception showed a very high F1-score on one of the folds for COOS, its high standard deviation led to a lower position in the final ranking compared to the more stable DenseNet121 and EfficientNetB0 (Table 3). The weights assigned to the metrics in the Score_adj_ formula reflect their clinical relevance. The F1-score (W = 0.35) was prioritized due to its balanced consideration of precision and recall, critical for accurately identifying high- and low-quality sutures. Precision and recall were each weighted at 0.20 to minimize false positives and negatives. AUC-ROC (W = 0.15) evaluates the model’s discriminative ability across thresholds, while accuracy (W = 0.10) serves as a secondary measure due to its lower sensitivity to class imbalance. The Score_adj_ metric also accounts for model stability across folds, reducing overfitting risks given the limited dataset.

In contrast to the work of Mansour et al. [26], which utilized synthetic polymer pads, our study employed real ex vivo tissue samples to enhance clinical relevance. The best models according to F1-score in their study were: Xception (95%), MobileNet (91%), and DenseNet (90%). Our results align with their findings but extend them to more realistic surgical scenarios. We also compared a larger number of CNN models and applied cross-validation for reliable assessment. Furthermore, the substantial inter-rater reliability (κ = 0.60–0.80) underscores the robustness of our ground truth labels, aligning with best practices for minimizing annotation subjectivity in medical imaging studies.

We selected state-of-the-art CNNs known for their performance in medical imaging tasks [19,20,21,22]. While no single model outperformed others across all suture types, ResNet50V2, DenseNet121, and Xception consistently ranked highest in both F1-score and stability-aware metrics. For COOS, DenseNet121 showed the best results in terms of F1-score and the adjusted weighted performance score (Score_adj_) (Table 3). Meanwhile, for other datasets with more visually homogeneous characteristics (IOVS and ILS), ResNet50V2 emerged as the leader in F1-score and/or Score_adj_ (Table 2 and Table 4). Xception also demonstrated high performance, particularly for vascular sutures (Table 4). EfficientNetB0 and MobileNetV3Large models demonstrated competitive results and high stability (low standard deviation) on most datasets, making them attractive candidates, especially considering their computational efficiency. Models from the VGG family (VGG16, VGG19), despite their simpler structure and popularity, often underperformed more modern architectures, especially in terms of F1-score, which may be due to their lesser ability to capture complex spatial feature hierarchies.

A notable finding was the relatively lower classification performance for COOS, where the maximum F1-score reached only 0.79, compared to over 0.93 for other suture types (Table 3). This performance gap likely stems from specific visual challenges inherent to this suture type that test the limits of our transfer learning approach. Unlike interrupted sutures (IOVS and ILS), where discrete stitches and knots provide clear, repeating features for analysis, the thread in a COOS is often continuous and partially embedded within the tissue. This results in less visible and more ambiguous patterns, making it difficult for the models to assess key quality indicators such as stitch uniformity and tension.

The challenge is exacerbated by the nature of the pre-trained models. Our use of weights from ImageNet-1K, a dataset of 1.28 million natural images across 1000 object categories, is a critical aspect of our methodology. While this approach proved effective for IOVS and ILS, the hierarchical features learned from ImageNet (e.g., distinct edges, textures, and object shapes) are less suited for identifying the subtle, fine-grained textural variations of a continuous suture line against biological tissue. The GradCAM visualizations (Figure 5) confirm that the models focused on suture lines; however, for COOS, these lines are inherently less distinct, which likely explains the performance drop. The model may struggle to differentiate a well-formed continuous suture from a poorly executed one where the thread is either too loose or too deeply buried, as both scenarios lack the strong visual cues present in other suture types. This suggests that the domain shift between natural images and the specific visual characteristics of COOS is a key limiting factor.

Our study utilized ex vivo porcine vascular and intestinal tissues to standardize data collection and generate a controlled range of suture qualities. While this approach provided biomechanical relevance and ethical practicality for proof-of-concept validation, the limited dataset size (100–190 images per suture type) and absence of in vivo human tissues constrain immediate clinical generalizability. Specifically, the exclusion of cutaneous sutures—where skin tone diversity critically influences visual features—limits applicability to diverse patient populations. Nevertheless, our methodology offers significant value in educational contexts: models trained on ex vivo substrates are currently being piloted at our institution to objectively assess suture quality in student training, thus reducing subjectivity in skill evaluation. Future clinical translation requires validation on annotated human tissue datasets encompassing varied anatomies and pigmentation. To mitigate domain shift, we maximized realism by using fresh ex vivo tissues with biomechanical properties mimicking live human tissue and employing expert-defined clinical criteria for annotation. Despite these measures, further validation in live surgical settings remains essential.

We acknowledge that our annotation protocol, in which disagreements between two primary experts were resolved by a third senior surgeon, represents a limitation. While this approach ensured consensus and conserved clinical resources, future studies could benefit from involving a larger panel of surgeons to evaluate all images independently, thereby strengthening validity through majority voting or weighted consensus.

Future work should prioritize advanced technical and methodological refinements. First, enhancing COOS classification could involve advanced pre-processing techniques such as contrast-limited adaptive histogram equalization (CLAHE) or frequency-domain filtering to improve suture thread visibility. Architectures with self-attention mechanisms or hybrid networks combining CNNs with handcrafted feature extractors may better capture subtle textural patterns in continuous sutures. Domain-specific pre-training on large medical imaging datasets before fine-tuning could further mitigate domain shift from ImageNet. For granular assessment, transitioning to ordinal scales (e.g., 1–5 quality tiers) using ordinal loss functions or regression CNNs would better reflect clinical skill gradients. However, this demands expanded datasets, rigorous annotation protocols to address inter-rater variability, and robust uncertainty quantification to ensure reliability in real-world applications.

## 5. Conclusions

This study demonstrated that applying transfer learning with pre-trained CNNs is an effective method for the binary classification of surgical suture quality from images. A comparative analysis of eight modern architectures showed that ResNet50V2, DenseNet121, and Xception models were most stable by Score_adj_ and achieved the best results in terms of F1-score, often exceeding 0.90 on laparoscopic and vascular sutures. While COOS classification (F1 = 0.79) revealed domain-shift challenges. These results confirm that transfer learning is a powerful strategy for developing automated surgical skill assessment systems, particularly in settings with limited annotated data. This approach provides an objective and standardized tool to enhance surgical training and quality control. Future work should expand datasets and develop models for a more comprehensive scoring-based assessment of suture quality.

## Figures and Tables

**Table 1:** Key metrics used for CNN performance evaluation.

Metric	Formula	Description
Precision	$\frac{T_{p}}{T_{p}+F_{p}}\cdot 100\%$	Proportion of correct positive predictions relative to all positive predictions.
Recall	$\frac{T_{P}}{T_{P}+F_{N}}\cdot 100\%$	Proportion of correct positive predictions relative to all actual positive cases.
F1-score	$\frac{2\cdot p\cdot r}{p+r}\cdot 100\%$	Harmonic mean of precision and recall.
AUC-ROC	-	Area under the curve that illustrates the trade-off between model sensitivity and specificity at different classification thresholds. The value ranges from 0.5 (random guessing) to 1 (perfect model).

Notes. *T_P_*—true positives, *T_N_*—true negatives, *F_P_*—false positives, *F_N_*—false negatives, *p*—precision, *r*—recall. In machine learning, the rarer event is typically considered the positive case, and the more frequent event is considered the negative case.

**Table 2:** Summary performance metrics of models for IOVS classification (mean ± std. dev. over 5 folds).

Architecture	Precision	Recall	F1-Score	AUC-ROC	Threshold	Score_adj_
External View						
Xception	0.902 ± 0.063	**0.983 ± 0.021**	**0.939 ± 0.033**	0.910 ± 0.067	0.374 ± 0.102	0.913 **
ResNet50V2	**0.917 ± 0.036**	0.948 ± 0.032	0.932 ± 0.031	**0.946 ± 0.025**	0.482 ± 0.074	0.915 *
MobileNetV3Large	**0.926 ± 0.053**	0.939 ± 0.035	0.932 ± 0.033	0.931 ± 0.039	0.476 ± 0.017	0.911 ***
VGG19	0.917 ± 0.058	0.930 ± 0.052	0.923 ± 0.046	0.915 ± 0.064	**0.484 ± 0.010**	0.895
VGG16	0.909 ± 0.041	0.922 ± 0.043	0.914 ± 0.013	0.914 ± 0.043	0.476 ± 0.027	0.905
DenseNet121	0.894 ± 0.047	0.922 ± 0.017	0.907 ± 0.018	0.885 ± 0.023	0.470 ± 0.060	0.892
InceptionV3	0.877 ± 0.090	0.939 ± 0.035	0.903 ± 0.040	0.886 ± 0.061	0.462 ± 0.104	0.876
EfficientNetB0	0.886 ± 0.034	0.922 ± 0.084	0.901 ± 0.037	0.918 ± 0.032	**0.580 ± 0.130**	0.882
Internal View						
ResNet50V2	**0.914 ± 0.017**	0.950 ± 0.032	**0.931 ± 0.011**	**0.959 ± 0.008**	0.334 ± 0.242	0.930 *
VGG16	0.897 ± 0.017	**0.960 ± 0.037**	0.927 ± 0.022	0.952 ± 0.030	0.382 ± 0.136	0.918 **
Xception	0.904 ± 0.030	0.940 ± 0.037	0.922 ± 0.033	0.930 ± 0.040	0.516 ± 0.060	0.904 ***
DenseNet121	0.886 ± 0.073	0.940 ± 0.058	0.909 ± 0.038	0.936 ± 0.034	0.458 ± 0.040	0.893
EfficientNetB0	0.886 ± 0.040	0.910 ± 0.066	0.896 ± 0.032	0.931 ± 0.036	0.394 ± 0.160	0.884
MobileNetV3Large	0.906 ± 0.085	0.890 ± 0.097	0.889 ± 0.026	0.928 ± 0.017	0.432 ± 0.056	0.885
VGG19	0.850 ± 0.053	0.940 ± 0.037	0.892 ± 0.036	0.890 ± 0.035	0.430 ± 0.135	0.872
InceptionV3	0.831 ± 0.071	0.930 ± 0.060	0.874 ± 0.039	0.866 ± 0.058	**0.478 ± 0.039**	0.852

Notes. Architectures are sorted in descending order of the mean F1-score. The best values in each metric column are highlighted in bold. In the “Threshold” column, the value with the smallest standard deviation (most stable classification threshold) is marked in bold. Asterisks (*, **, ***) denote 1st, 2nd, and 3rd ranked models per Score_adj._

**Table 3:** Summary performance metrics of models for COOS classification (mean ± std. dev. over 5 folds).

Architecture	Precision	Recall	F1-Score	AUC-ROC	Threshold	Score_adj_
DenseNet121	0.825 ± 0.128	0.805 ± 0.165	**0.788 ± 0.071**	**0.853 ± 0.080**	0.470 ± 0.055	0.787 *
Xception	0.738 ± 0.264	0.810 ± 0.048	0.740 ± 0.153	0.842 ± 0.108	0.340 ± 0.157	0.700
EfficientNetB0	0.845 ± 0.142	0.676 ± 0.107	0.734 ± 0.055	0.823 ± 0.054	0.368 ± 0.078	0.746 **
MobileNetV3Large	0.831 ± 0.164	0.681 ± 0.085	0.730 ± 0.042	0.829 ± 0.047	0.394 ± 0.125	0.745 ***
VGG16	**0.925 ± 0.150**	0.619 ± 0.142	0.716 ± 0.078	0.781 ± 0.069	**0.486 ± 0.015**	0.729
ResNet50V2	0.756 ± 0.244	0.743 ± 0.076	0.715 ± 0.116	0.809 ± 0.108	0.396 ± 0.163	0.692
VGG19	0.626 ± 0.037	0.771 ± 0.172	0.685 ± 0.092	0.811 ± 0.057	0.306 ± 0.117	0.674
InceptionV3	0.644 ± 0.235	**0.814 ± 0.138**	0.676 ± 0.108	0.752 ± 0.100	0.342 ± 0.115	0.662

Notes. Architectures are sorted in descending order of the mean F1-score. The best values in each metric column are highlighted in bold. In the “Threshold” column, the value with the smallest standard deviation (most stable classification threshold) is marked in bold. Asterisks (*, **, ***) denote 1st, 2nd, and 3rd ranked models per Score_adj_.

**Table 4:** Summary performance metrics of models for ILS classification (mean ± std. dev. over 5 folds).

Architecture	Precision	Recall	F1-Score	AUC-ROC	Threshold	Score_adj_
ResNet50V2	**1.000 ± 0.000**	0.908 ± 0.084	**0.950 ± 0.047**	0.959 ± 0.036	0.428 ± 0.081	0.931 *
VGG16	**1.000 ± 0.000**	0.903 ± 0.145	0.942 ± 0.089	0.969 ± 0.045	0.356 ± 0.093	0.911
DenseNet121	**1.000 ± 0.000**	0.903 ± 0.145	0.942 ± 0.089	0.957 ± 0.061	0.454 ± 0.032	0.908
EfficientNetB0	0.931 ± 0.057	0.953 ± 0.058	0.941 ± 0.053	**0.971 ± 0.024**	0.488 ± 0.125	0.920 **
MobileNetV3Large	0.964 ± 0.073	0.928 ± 0.099	0.940 ± 0.056	0.965 ± 0.039	0.434 ± 0.089	0.919 ***
Xception	0.978 ± 0.044	0.858 ± 0.143	0.905 ± 0.079	0.920 ± 0.082	0.412 ± 0.047	0.876
InceptionV3	0.823 ± 0.116	**0.978 ± 0.044**	0.889 ± 0.073	0.945 ± 0.048	**0.472 ± 0.030**	0.866
VGG19	0.837 ± 0.111	0.956 ± 0.089	0.882 ± 0.048	0.924 ± 0.035	0.360 ± 0.165	0.872

Notes. Architectures are sorted in descending order of the mean F1-score. The best values in each metric column are highlighted in bold. In the “Threshold” column, the value with the smallest standard deviation (most stable classification threshold) is marked in bold. Asterisks (*, **, ***) denote 1st, 2nd, and 3rd ranked models per Score_adj_.

## Data Availability

The datasets, training notebooks, statistical analysis and supplementary visual results are available in the GitHub repository at https://github.com/mxsolopov/surgical-sutures-classification (accessed on 23 July 2025).

## Abbreviations

The following abbreviations are used in this manuscript:

CNN	Convolutional neural network
COOS	Continuous over-and-over open suture
ILS	Interrupted laparoscopic suture
IOVS	Interrupted open vascular suture
